# Community perspectives on ideal bacterial STI testing services for gay, bisexual, and other men who have sex with men in Toronto, Canada: a qualitative study

**DOI:** 10.1186/s12913-022-08529-7

**Published:** 2022-09-23

**Authors:** Jayoti Rana, Ann N. Burchell, Susan Wang, Carmen H. Logie, Ryan Lisk, Dionne Gesink

**Affiliations:** 1 MAP Centre for Urban Health Solutions, Li Ka Shing Knowledge Institute, St. Michael’s Hospital, Unity Health Toronto, Toronto, Canada; 2 Department of Family and Community Medicine, MAP Centre for Urban Health Solutions, Li Ka Shing Knowledge Institute, St. Michael’s Hospital, Unity Health Toronto, Toronto, Canada; 3 Dalla Lana School of Public Health, University of Toronto, 155 College St, 6th Floor, Toronto, Canada; 4 Factor-Inwentash Faculty of Social Work, University of Toronto, Toronto, ON M5T 3M7 Canada; 5 ACT (AIDS Committee of Toronto), Toronto, ON Canada

**Keywords:** Bacterial STI, Clinical intervention, GBM, STI testing

## Abstract

**Background:**

Innovation is needed to produce sustained improvements in bacterial sexually transmitted infections (STI) testing given suboptimal access and uptake among sexually active gay, bisexual or other men who have sex with men (GBM). Yet, the STI testing processes and technologies that best address local testing barriers among GBM in Toronto is unknown. We aimed to explore men’s perspectives regarding STI testing services for GBM to identify and prioritize new STI testing interventions in Toronto, Ontario, Canada.

**Methods:**

We conducted four focus groups with twenty-seven GBM in 2017: two with cisgender men living with HIV, one with cisgender HIV-negative men, and one with transgender men. Twenty-seven men participated in the focus groups with 40% 18–30 years of age, 48% self-identifying as white, and the remainder self-identifying as Middle Eastern, Latino/Hispanic, Asian/Pacific Islander, South Asian, First Nations, African/Caribbean/Black, or mixed race. 59% of participants self-identified as living with HIV. Participants were asked about their STI testing experiences in Toronto, barriers and facilitators to testing, and ideal STI testing process. Focus groups were audio recorded, transcribed verbatim, and analyzed using thematic analysis.

**Results:**

Core concepts included how clinical context, bacterial STI testing delivery, and interactions with healthcare providers can create barriers and recommendations for ways to improve. Regarding clinical context, participants desired more clinics with accessible locations/hours; streamlined testing that minimized use of waiting rooms and wait times; and improved clinic ambience. Bacterial STI testing delivery recommendations included standardization to ensure consistency in sexual history intake, tests offered, follow-up and public health reporting between clinics. Men also recommended reducing the multistep process testing by offering components such as lab requisitions and results online. Participants also recommended interactions with healthcare providers be professional and non-judgmental, offer compassionate and competent care with destigmatizing and lesbian, gay, bisexual and trans (LGBT) affirming communication.

**Conclusion:**

Concrete and practical solutions for improving existing sexual health services and facilitating optimal STI testing include streamlining testing options and providing patient-centred, LGBT-affirming care to enable optimal STI testing.

## Background

Bacterial sexually transmitted infections (STIs) were increasing substantially worldwide pre-COVID-19. Gay, bisexual and other men who have sex with men (GBM) shared a disproportionate burden of STIs in urban centres across North America, including Canada [1–4]. Current Canadian clinical guidelines recommend sexually active GBM receive bacterial STI tests at minimum once per year or every three months if at ongoing risk (e.g., new or multiple sexual partners, anonymous or casual sexual partners, having unprotected sex, or use of substances before or during sexual encounters) [5]. Implementation of these guidelines apply the ‘test and treat’ prevention and control principles to mitigate adverse health outcomes at the individual level and reduce transmission at the population level. However, STI testing remained below recommended guidelines among GBM in Toronto—Canada’s largest city (Population: 2.7 M), which has both a large GBM population and epidemic rates of syphilis and gonorrhea [6, 7].

In Toronto, STI testing services are offered in primary care practices, specialist services and dedicated sexual health clinics. Sexual health clinics are unique because they do not restrict services based on residency status [8]. Primary care practices are located throughout Toronto, while the few sexual health clinics and specialist services are mostly located in the downtown core [8]. Prior to the COVID-19 pandemic, men had to see a healthcare provider in person to access STI testing services. That healthcare provider would conduct an interview, obtain a risk assessment, and evaluate STI risk factors and sexual practices to inform counseling and testing recommendations. According to Canadian clinical guidelines, bacterial STI testing should reflect organism and exposure [5]. As such, bacterial STI testing includes first void urine sample and extragenital swabs (i.e., pharyngeal and rectal swabs) for chlamydia and gonorrhea, and serology for syphilis. Unlike testing for HIV, there are currently no anonymous testing options for bacterial STIs in Toronto.

Past efforts to increase STI testing uptake among GBM in Toronto include a “testing blitz” in 2011–2012 run by the Ontario HIV Treatment Network, in partnership with Toronto Public Health, called “Get It On”. This strategy involved intensive online and offline messaging combined with increased testing capacity at the largest sexual health clinic in Toronto. “Get It On” achieved a 20% increase in STI testing among GBM in Toronto. Much of the increase in testing was attributed to greater testing capacity created by adding more clinic sites and hours for drop-in testing [9]. However, in addition to number of people tested, frequency of testing is essential to achieving effective control of bacterial STI rates [10]. Thus, testing blitzes alone are inadequate strategies for improving STI testing.

Following the “Get It On” testing blitz, there were minimal changes in how STI testing services were delivered in Toronto. Studies have identified lack of access to timely and convenient STI testing services, privacy and confidentiality concerns, lack of knowledge at the provider or patient level, lack of appropriate inclusive language, and stigma as barriers to STI testing for GBM [11–15]. With advances to testing, communication, and healthcare delivery, innovation is needed to produce sustained improvements in bacterial STI testing uptake and access among GBM. GBM may be more likely to access bacterial STIs testing, and test more often, if testing processes and technologies are appropriate, acceptable, and preferred. Yet, we do not know which STI testing processes and technologies would best address testing barriers among GBM in Toronto. Therefore, our objective was to explore reasons why GBM in Toronto may or may not get tested for bacterial STIs, and what would make testing acceptable in order to inform the design of bacterial STI testing interventions and improve testing uptake.

## Methods

This qualitative study emerged from a pragmatic paradigm [16]. We took a solutions-based approach to identify actionable solutions to address our objective of how to improve STI testing uptake among GBM in Toronto. We used thematic analysis [17] of focus groups to explore the current and ideal bacterial STI testing process experienced by GBM in Toronto. This study was conducted in Toronto, Canada, because it has a large population of GBM and bacterial STI epidemics (syphilis and gonorrhea) among this population. Participants were recruited and focus groups were held between August to December 2017. Unity Health Toronto and University of Toronto research ethics review board reviewed and approved this study.

### Participants and data collection

GBM were purposively sampled to ensure diversity in age, race, sexual and gender orientation (i.e., identify as a cis or trans gender man), and HIV status. Potential participants were enrolled by a peer recruiter from ACT (formerly the AIDS Committee of Toronto), an HIV/AIDS service organization with extensive experience working with diverse groups of GBM in Toronto and trusted by the community. Recruitment flyers were sent out across the professional network of ACT contacts and posted on social media. Potential participants called or emailed the peer recruiter to indicate their interest in the study and were provided an information sheet about the study. Eligible participants included men who self-identified as adult GBM (18 years old or older), who lived, worked, or otherwise spent time in Toronto, were able to converse in English, and were comfortable talking about STI screening.

A semi-structured interview guide was informed by the literature [13, 18] and developed by members of the team, which included researchers involved in promoting, providing, and evaluating sexual health promotion including STI testing to GBM (ANB, DG, CHL), and one team member from ACT (RL). The interview guide was used to ask participants about: past experiences with bacterial STI testing; barriers and facilitators to bacterial STI testing; and what an ideal bacterial STI testing experience would look like from beginning to end. Participants also self-administered a brief demographic questionnaire after the focus group.

Four focus groups were conducted in groups of three to 10 participants each: two groups comprised HIV positive cisgender men (*n* = 16), one group comprised HIV negative cisgender (*n* = 8) men, and one group comprised HIV negative transgender men (*n* = 3). A second focus group comprised of HIV positive cisgender men was required as data saturation had not been achieved with the first group. Twenty-seven men participated in the focus groups. 40% were 18–30 years of age, 30% were 30–50 years of age, and 30% were over 50; all trans-identified men (*n* = 3) were under 30. 48% of men self-identified as white, and the remainder self-identified as Middle Eastern, Latino/Hispanic, Asian/Pacific Islander, South Asian, First Nations, African/Caribbean/Black, or mixed race. Over half (59%) of participants self-identified as living with HIV. One participant shared additional information after the focus group they participated in since they had withheld some comments during the focus group in efforts not to be disruptive to the group dynamic or dominate the conversation. These additional comments were included in the analysis.

All focus groups were conducted anonymously and facilitated by two research team members: the discussion was led by a middle-aged, heterosexual, cisgender woman, who had experience leading focus groups on the social epidemiology of sexual health (DG); and supported by a middle-aged, gay, cis-gender man, who had experience providing sexual health counselling, resources, and other services to the community (RL). The lead investigator, a middle-aged, heterosexual, cisgender woman who had experience leading quantitative sexual health research (ANB), was also present during focus groups in case additional questions needing exploration emerged during the discussion. All focus groups were conducted at ACT, in downtown Toronto; lasted approximately 90–120 min; were voice-recorded, transcribed verbatim, and verified for accuracy. Field notes documented non-verbal communication during each discussion. Participants were compensated $40 for their time, knowledge, and transportation costs.

### Data analysis

We used thematic analysis [17] to inductively describe the overarching themes of barriers to bacterial STI testing; facilitators of bacterial STI testing; and ideal STI testing experiences. Data saturation for the analysis was reached with completion of the fourth focus group. Transcripts were read and re-read, along with fieldnotes; then coded using open coding by two cisgender female research assistants (JR, SW). Open codes were reviewed and discussed for focused coding by the research team, which included the lead investigator (ANB), two facilitators (DG, RL), one queer cisgender woman with a research program in queer sexual health (CL), and one gay cisgender man with a research program in HIV prevention (LN). Focus groups were recoded using focused coding and grouped, along with pertinent quotes, into broad categories within the predetermined themes. The research team met again to review and interpret findings.

Qualitative research rigor was addressed by involving a diverse group of expert researchers and community members in the design, data collection, analysis, and interpretation of the study (investigator triangulation) [19, 20]; collecting data anonymously and with clear discussion group ground rules centered around respect to ensure psychological safety of participants; using an iterative data analysis process; providing ample description to support interpretations; and regular reflexivity, which included responding to power differentials and knowledge gaps identified that might affect data collection, analysis, or interpretation.

## Results

### Accessing bacterial STI testing in Toronto

Participants accessed bacterial STI testing services from sexual health clinics, walk-in clinics, primary care clinics, or their HIV care providers in Toronto. Many preferred their primary care physician compared to sexual health clinics because of established and positive patient physician relationship, which involved active listening and good communication. Conversely, some participants did not have a primary care physician, and highlighted difficulties in finding a provider they were comfortable with and was accepting new patients.

Motivating factors for seeking bacterial STI testing included: notified by a partner they had an STI; experiencing symptoms; or experiencing a risky sexual encounter, such as “stealthing”, (non-consensual condom removal). Participants taking pre-exposure prophylaxis for HIV prevention and HIV-positive participants described routine STI testing as part of their routine care. Otherwise, participants indicated barriers that prevented regular bacterial STI testing. In keeping with a pragmatic paradigm, we organized our results to follow the logic of a clinic workflow, highlighting barriers and solutions at each stage of bacterial STI testing delivery. Participants described barriers and recommended ways to improve testing related to clinical context, test delivery, and interactions with healthcare providers.

### Clinical context

#### Geographical location & clinic hours

The first step to accessing bacterial STI testing is finding a clinic. Participants highlighted a lack of sexual health clinics in Toronto (Table 1). Moreover, some were difficult to access because they were not “on a major transit route.” (P5 extended interview, FG1). One participant shared some clinics required a “postal code registered in the area” (FG4, P2) to access services. Participants described clinics only open during “day hours” (FG1, P7) as inconvenient for those working or attending school and noted limited options to test during the weekend (Table 1). One participant described that despite having a family doctor who provided culturally competent and non-stigmatizing care, the long distance needed to travel prevented routine testing (Table 1). Some participants spoke of a lack of sexual health or walk-in clinics welcoming transgender and gender non-conforming individuals (Table 1). They described needing to act cisgender, “identify[ing] as something what you’re not” (FG3, P2), to access testing services.

**Table 1:** Barriers and recommendations to improve bacterial STI testing: clinical context

Subtheme	Barriers	Recommendations	Supportive Quotations
Geographical Location & Clinic Hours	Too few clinics inclusive to all men, limited hours of operation, and long distance needed to travel to clinics	More sexual health clinic locations inclusive to all men across the city with evening and weekend hours and accessible by transit	“There’s no way you can get tested on a Saturday night, you know, like, and then you’ve gotta make an appointment, go in on the Monday.” (FG1, P6) “I do [have a good family doctor], I haven’t seen him in a really long time because it’s hard for me to get down here.” (FG3, P3) “[The drop-in clinic] is not a very inclusive or safe feeling space for me. For trans people. So I don’t want to go to my local drop-in clinic.” (FG3, P3) “I tend to play the gender fluidity to my advantage […] it’s easier to get away with gender fluidity, identify as a woman, so I would enter a lesbian space for safety. […] any time I call, go to a clinic, I would play up the femininity, I would change, like just, because I was like, I have to fit in somewhere right now so, I’m gonna pretend to be a cis woman for this entire interaction.” (FG3, P2) “A lot of people I can only imagine like, if they’re working or have a job or are a full-time student, or I don’t know. Lots of things. Yeah. Accessibility. And the hours are like, 5 pm, 6:30. Please note, arrive before 4:30 pm. I don’t know. But like accessibility [of clinic hours] is important.” (FG4, P2) “I still feel like we’d constantly choose the Village, but gays are all over the city.” (FG1, P7) “Something close to transit, I would think, close to subway stations, […] and it doesn’t have to be subway stations, it can be like, rapid bus lines.” (FG1, P5 extended) “This is for testing for people assigned female at birth or something like that instead because like, then you know, it feels more welcoming to trans people because you’re just like, oh well, if they’re saying, assigned female at birth, then they know that, you know, trans, that like, then they must be inclusive of trans people.” (FG3, P1)
Waiting Room	Lack of anonymity	Streamlining testing by limiting time spent time in waiting room with a call back notification system and using numbers instead of names to call people from the waiting room	“Especially cause, as you mentioned too, it’s also a very small community and like being in that room and then like you’re sitting down, and you’re like, hey, I had sex with that guy last week. Or seeing your ex and their new boyfriend there.” (FG2, P1) “The fact that there’s so many people there, where you check in, and where they possibly will ask you personal questions, people can hear the questions. They can hear them ask. They can hear what your response is.’ (FG4, P6) “P5: It’s not anonymous P1: Nobody needs to know my business. It becomes you know what, I’m broadcasting it to whoever’s in that room at the time. And like you said, that room is full.” (FG4) “The waiting room situation that someone mentioned, like people have to like sit and look at each other as opposed to like, you couldn’t just do one person in a booth or have a set-up time, just like, go in, go out, no one needs to know that you’re getting tested.” (FG1, P7) “Like I think what they should do is give you a number basically or you know, one of those vibrating, you know, things like you’re in a restaurant.” (P1, FG4) “I just think like you could always do like, like a leave a number basis […] you literally walk in, you fill out the form, you leave a number, and you walk out, and then they just like ring you, […] rather than you’re just like sitting in a room” (P4, FG2) “The ideal would be, I walk up to a counter and say hi, I think I have a problem, and they’d say OK, have, have a seat, somebody will be with you right away, somebody taps you on the shoulder or calls your number or whatever, I’d prefer not a name, so that you’re not broadcasting to anybody else.” (FG4, P1)
Waiting Time	Long wait times	Streamline testing by focusing on specimen collection, including self-collecting samples, and proceeding to a lab directly to provide samples to reduce wait times	“Like the fact that the wait times are steeply climbing, like no matter what time you go, at the end of the period clinic, when, at the beginning, the testing period, it’s always like half an hour to two hours wait so I’ve just kind of like, very shied away from that now.” (FG2, P5) “P6: And just to touch on his point, when you were talking about when you come in and it’s, there are actually people sitting there like, a half hour before they even technically open. That’s how busy it is P1: Yep. Like when they switch over between, the men’s clinic and the female clinic, if you, if you’re a male and you go in during the female clinic, and you sit down, you register and you sit down. Cause then when it switches over, you’re way ahead of the lineup P2: It’s like camping for Black Friday discounts.” (FG4) “My [family] doctor is very booked […] I’m not gonna sit with gonorrhea […] for weeks and weeks, just to see him.” (FG4, P5) “They [those going to their family doctor] end up getting tested […] closer to once a year or less when they’d be willing to be tested 3 times a year if the services were available.” (FG1, P5 extended) “And then, Church and Wellesley, [which offers inclusive care to all men] was like, oh you have to be on like a 9-month waiting list” (FG3, P2) “All they wanted was a urine sample. Just a urine sample! I could’ve easily done that, no. It was sit down, wait, like, and, it was like 50 people ahead of me. Like it’s, you know, and I, and then they give me the, the thing, [urine specimen container], and I was like, they were like washroom’s right there, just leave it in the basket, and I was like, see how easy that could have been? It could have just been like boop, done. Leave.” (FG4, P5) “At least nowadays with my doctor, if I call him and say I have the following thing, the doctor will pre-do the requisition, and you just go pick it up.” (FG4, P1) “Why can’t there be something where I walk into a laboratory and say listen, I’d like to do an STI test and they know what to do. And they’ll say to you, well have you done x, y, and z today? Have you done this, you know, or here’s the sheet of paper, come back tomorrow. And, it’s far easier to go to a laboratory nowadays and, and do a test than it is to go to [a clinic]. (FG4, P1)
Clinic Ambience	Uncomfortable, overwhelming and anxiety provoking environment. Less accessible to non-English speakers and ethnoracial minorities	Non-judgmental safe environments with posters that emphasize inclusive spaces and information available in multiple languages	“Because it is fairly identity based, most of the people there are gay, or trans, and most of the volunteers present as such, which is fine, but because there’s not a lot of other resources, when there is like a straight man or, let’s, even better, an MSM who’s on the down-low, showing up there, it’s really, it can be really uncomfortable for them.” (FG1, P5 extended) “Like that moment when you’re entering and all these gay eyes are like staring at you, I, I can echo that feeling of, super-uncomfortable. […] And I can only imagine for the poor 18-year-old kid, first time, […] he gets through that door and he’ll have these like 50 gays guys like looking at him, or not gay or, whatever, […] people just like look at you, no matter, it doesn’t have to be judging, they just, curious.” (FG2, P5) “It’s important, that it [information] should be in different languages (FG2, P1) “That’s why I don’t go there because like, if I sit next to like, all these guys and some of them are not reading me as male, then doing the men’s hours, like, yeah, how will I sit there, right? Cause like, half of them do read me as male, half of them don’t.” (FG3, P2) “One tip I did hear that was really useful is that, having hospital spaces already have posters of trans folks, in campaigns cause then it just kinda gives like, while you’re in the waiting area, that this is a trans friendly space […]so that kind of makes people feel comfortable” (FG3, P2)

To address these barriers, participants suggested “there should be more places that we can go to if you actually want to walk in and visit” (FG4, P3), including “pop-up shops” (FG1, P7). Participants endorsed having more sexual health clinic locations around the city (not only in the downtown core) that offer drop-in services with evening and weekend hours (Table 1). One participant highlighted the need for accessibility by transit and “if I Googled STI clinic in Toronto […] I got a clear website with a good number of clinics” (FG1, P5 extended). Trans men highlighted clinics need to be inclusive of all men regardless of gender assigned at birth (Table 1).

#### Waiting room

Participants viewed spending time in the waiting room as a necessary but uncomfortable step to bacterial STI testing. Many found the waiting room of sexual health clinics in the gay village lacked anonymity because the GBM community is small (Table 1). Participants spoke about running into individuals they met in social settings or ex-partners. One participant expressed concern conversations with the receptionist were heard by everyone sitting in the waiting room, further compromising confidentiality (Table 1).

Participants’ recommendations for improvement involved streamlining testing and decreasing time spent in the waiting room. They preferred attending clinics at a scheduled time and being placed into a private room on arrival (Table 1). Others wondered if the “give you a number” model used when waiting for a table at a restaurant could be applied in the clinic setting (Table 1). Participants also suggested using numbers, instead of names, when calling individuals to the reception desk to preserve confidentiality in the waiting room (Table 1).

#### Waiting times

Participants described sexual health clinics as being very busy, with worsening wait times for drop-in clinics, some “up to 4 h”. Clinics were “so packed with standing room only” (FG4, P5). A sexual health clinic offering after-work hours had “people sitting there like, a half hour before they even technically open.” (FG4, P6). This led many to turn to their primary care provider instead for bacterial STI testing. However, it can be difficult to see a family doctor in a timely manner and can lead to less frequent testing (Table 1). Trans men shared clinics known to serve transgender and gender non-conforming persons had long patient wait lists (Table 1).

Participants perceived inefficiency in long wait times that could be solved. Clinics offering self-collection of samples were perceived as more efficient (Table 1). One participant described how his primary care doctor streamlines the bacterial STI testing process: “You show up at the allotted time, you’re in [to see the doctor], then you’re passed off to a nurse, […] whether it’s pee in a cup or whatever […] and you’re in and out, you’re not sitting in a waiting room for 2 h.” (FG2, P6). Additional suggestions for streamlining testing and decreasing wait times included accessing a lab directly for testing, with a few participants already receiving lab requisitions directly from their primary care provider (Table 1). Participants strongly supported self-initiated online access to lab requisitions: “it could be great to be able to print your own” (FG4, P3).

#### Clinic ambience

Clinic ambience can enhance or erode the testing experience. It can be a barrier if it is experienced as uncomfortable and overwhelming to the senses and emotions, especially for those uncomfortable discussing their sexuality (Table 1). Participants described clinic environments inducing anxiety with everyone “staring” (FG2, P5) at each other in the waiting room, and expressed concern that this may prevent first time or younger patients from coming in to get tested (Table 1). Participants also shared concerns clinics were not welcoming to ethno-racial minorities, newcomers, and persons who speak a first language other than English. “There’s language barriers, there’s this public healthcare, I do feel like it’s very white when you go.” (FG1, P7).

Recognizing bacterial STI testing can be an awkward or distressing experience, especially for first time testers, men suggested clinics must be “a place that’s welcome, non-judgmental, where people feel easy and safe.” (FG4, P3). Participants discussed “how stigmatisation is different between and within cultures” and the role of “culturally, contextually appropriate setting[s]” (FG2, P4) to promote bacterial STI testing. Information in languages other than English would also be helpful (Table 1).

Some sexual health clinic services are separated by gender, which caused confusion and anxiety for trans participants since they were unsure which clinic to attend and feared transphobic interactions in the waiting room (Table 1). One trans participants suggested using sex instead of gender, though also recognized this binary system could still be problematic, “Female assigned at birth or male assigned at birth helps. You know, because then you’re like, that’s what I was assigned, those are the body parts I have. […] When you do man/trans […] it excludes a lot of folks that identify as non-binary.” (FG3, P2). Additionally, participants endorsed posters in the waiting room including transgender and gender non-conforming language and images is a small but significant step to creating a more inviting and welcoming clinic space (Table 1).

### Bacterial STI testing delivery

#### Sexual history intake

Participants felt it was necessary to normalize questions about sexual activity and not make assumptions about who requires a test (Table 2). Many acknowledged some men are not aware that bacterial STIs can be asymptomatic, and rely solely on symptoms to seek testing. For example, “I’ve told him, [a sexual partner], you need to go get tested for gonorrhea. And his justification […is] I feel fine, I don’t have any of the symptoms” (FG4, P5). Participants also recognized the importance of taking a good sexual history because individuals may not necessarily know what they need. One participant described, “Sometimes I see a nurse and they ask me, what would you like to get tested for? And to me, I think that’s a very silly question. For me, it’s like, you’re an expert in sexual health.” (FG2, P4). Participants agreed standardizing sexual history questions to ask about sexual experiences enables testing that better reflects all possible experiences of individuals (Table 2).

**Table 2:** Barriers and recommendations to improve bacterial STI testing: bacterial STI testing delivery

Subtheme	Barriers	Recommendations	Supportive Quotations
Sexual History Intake	Making assumptions about who requires a test based on demographics or symptoms	Normalizing sexual activity questions in healthcare	“I think doctors have to do a better job of not making assumptions, when they’re dealing with people of a certain age or a certain background and still have that conversation about, maybe you should have a test.” (FG1, P3) “Another interesting thing is, if you are basing on male or female then it becomes like, sometimes they’ll ask you, who did you sleep with to get tested, at that time. So if you say, female, whether you’re like, what if I slept with a trans woman, what happens then? Right? I still am concerned with the same issues that I would, if I was sleeping with that male identified, or male partner, assigned at birth.” (FG3, P2) “P4: Maybe if you ask me, can you tell me your sexual experiences since you last came to this clinic, and I tell you, I did this and I did this and I did this and I did this, then the nurse, with that information says, you should be tested for this and this and this and this, and a pharyngeal swab and a rectal swab and this is this, so it’s the type of questions that are being asked that will really, make effective decision making happen. P3: Preach!” (FG2)
Testing Offered	Lack of consistency and clarity on testing	Providing verbal and written communication explaining tests needed by sexual exposures	“I’ve done STI testing through my family doctor and I say, I would say to doctor, test everything, I mean, syphilis, chlamydia, everything, and I just assumed that gonorrhea was part of it, was part of the blood work. […] because I just thought that the blood work or a urine, or a urine sample, could test whether you have gonorrhea in any part of the body.” (FG2, P2) “Break down what they’re testing you for cause a lot of the times you’re not being tested for what you think you’re getting. You’re getting tested for mostly chlamydia and HIV.” (FG2, P3) “Another issue too, as well as people not knowing how to get tested, as well, for certain people, cause it can be at the, at the site of infection, so like, depending whether you get like, doing like a pee test or if you need like a swab.” (FG4, P2) “Can you incorporate all of the symptoms […] into a one-page thing and say, these are the symptoms, these are, this is how you get tested. Like it’s not a one-day thing. You know, you’ve gotta go in, give blood, urine, whatever” (FG2, P6)
Follow-up & Test Results	Not provided test results	Clear communication regarding follow-up steps for testing, including sequence of events for a positive test and how results will be made available	“That’s another annoying thing is that, they don’t relate to timing for testing, is that they don’t call you if you’re negative. So you’re like waiting for a month.” (FG2, P5) “I think people should know, prior to going in, what the, the outcome, like what’s the set of procedures, what if you test positive? You know, and, you know, how is treatment, how are you going to get onto treatment, things like that.” (FG1, P6) “Maybe give me an information pamphlet on, OK, what, what do I do if I’m, if I test positive? Here is where you can go if you need support, end of story, and even access it online as well.” (FG4, P6) “I: Would it be helpful at all to be able to access your lab results online? P: Oh, yes I: Yes, yes, yes? From this end? Yes? P6: Yeah, so my ideal would be is that I can go anywhere, say I want to have this test done, give me whatever the cup I need to do, have it done, put it in the little box, leave, and be able to access the results online Ps: I agree with that.” (FG4) “P1: Like I mean even, even if it goes, the results go back to your doctor and your doctor contacts you, yeah. I mean, I don’t see a problem with that P5: That would be alright P1: I mean, that way your doctor is the best one to give you advice, give you medication P5: And medication.” (FG4)
Public Health Reporting	HIV non-disclosure criminalization, lack of clarity on use of case and partner information	Affirming confidential testing and clarifying how testing information is utilized by public health	“If you do test positive for anything, they had a, a social worker there. […] You had to meet with them right after. And, that scared me because it’s like oh, […] you’ve been in contact with other people, it’s your responsibility, so I kind of felt oh my god, now on top of what I’m doing here, I’ve got this big thing put on my shoulders now that I’m responsible for all of the other people that I’ve slept with. So I have to now contact everybody and are they gonna be monitoring who I contact? Or are they gonna know who I contacted? […] I felt that, you know what, they’re, they were controlling what I was gonna do, and who I was gonna be with.” (FG4, P1) “I also want to mention that like, there are a lot of undocumented folks here and access to care is really essential to this, folks don’t go to the doctor if they’re undocumented. A, they have to pay. And that’s a big barrier, at times. And B, like, they’re scared that the doctor might rat them out.” (FG2, P1) “So when you admit that you had unprotected sex and you already know, have been living with HIV, that’s a serious thing to admit. And then it becomes reportable to public health, like is that not a barrier?” (FG4, P5) “I prefer anonymous testing, because when you’re not anonymous in testing, they have to report it to Toronto Public Health and that really compromises my ability to control my own health.” (FG2, P1) “One thing my doctor used to do is any test, especially for HIV, is he never, the test would go in with a number. Not with a name. And he kept his own sheet, and if it came back positive, then that’s how he dealt with it.” (FG4, P1) “P5: What if we just provided like the phone number of the person who may have been in contact with Gonorrhea, and then they just get this beautiful text message saying, “Hey, you might want to like” … P2: Well Halton sends the text message… P4: Public Health does that. […] P2: Well, in Peele they send a text message P5: They send a message? P2: I’ll get a message that says someone listed you a contact for this, if you find time please come in and get tested… P4: And it’s totally anonymous P3: That’s not too bad then” (FG4)
Multi-step Process	STI testing causes stress due to compromised anonymity and social stigma	Streamlining testing process by accessing testing lab requestions, and results online	“Yeah, so my ideal would be is that I can go anywhere, say I want to have this test done, give me whatever the cup I need to do, have it done, put it in the little box, leave, and be able to access the results online. […] Maybe give me an information pamphlet on, OK, what, what do I do if I’m, if I test positive? Here is where you can go if you need support, end of story, and even access it online as well. (P6, FG4) I: So, something where you can have like, an online request, […] so you can request it and then they give you the paperwork. And then you can bring it to a lab, get your results, […] either through your doctor or online. Almost sounds like you could then have an extra couple of boxes, one that says where you want your prescription, if you’re positive, being sent to and you could click another box if you want counselling or some other support P: Yeah! P: Mm hmm I: With it. And then, ta da! P3: No, seriously, this is the twenty-first century, yeah. I mean … P4: New ATM machines I: Yeah, it’s kind of like the ATM of STD’s P3: If you can meet somebody to screw online, you can get treated P3: To me that should be the add (FG4)

#### Testing offered

Participants’ descriptions of testing services identified a lack of consistency and clarity on testing received, both with respect to the type of bacterial STI as well as the anatomic site of specimen collection. Some participants who believed they had received complete STI testing did not know which samples and tests were ordered for chlamydia, gonorrhea, and syphilis (Table 2).

To improve understanding, participants stressed the need to inform men about the kinds of sexual exposures that would prompt a need for a particular test (Table 2). One participant explained: “As soon as I learned about the swabbing of the throat for gonorrhea, *everybody* heard that. I told everyone who was willing to listen […] I felt empowered.” (FG2, P3) An easy-to-read one-page fact sheet for each bacterial STI to distribute in clinics or provide online was also suggested to facilitate consistent information sharing regarding when each bacterial STI test is offered (Table 2).

#### Follow-up & test results

Many clinics take a “no news is good news” (FG4, P5) approach to providing bacterial STI test results, such that men were only contacted if their test was positive. However, this practice “creates an anxiety for people.” (FG4, P2). Participants believed test results, whether positive or negative, should be communicated to testers in a timely way (Table 2). Many participants felt it was important to standardize follow-up procedures, so men know how they will receive test results (Table 2). Some men felt a pamphlet outlining steps was needed and were hopeful these pamphlets could be made accessible online (Table 2). Furthermore, participants supported the idea of being able to see test results for themselves on an online platform, along with copy sent to their primary care doctor (Table 2).

#### Public health reporting

Bacterial STI cases are reportable to public health for surveillance and partner notification purposes. Partner notification can occur one of three ways: the case notifies partners, the physician notifies partners, or public health notifies partners [21]. For men living with HIV, public health reporting of bacterial STI cases and partner notification was concerning as HIV non-disclosure is criminalized in Canada. One HIV positive participant shared, “some people have avoided getting STI tests because they’re afraid of legal consequences. I don’t, I’m not suggesting that’s in the broad spectrum, but I have met some people who have that, that fear” (FG1, P3).

Moreover, participants shared they were uncertain how case and partner information was used by public health. One HIV positive participant expressed concern about relational accountability, stating the reporting process made them feel as though they were being watched and their freedom was taken away (Table 2). One participant described how undocumented individuals with precarious immigration status, such as refugee claimants or those without permanent resident status, may not access testing because they fear providers will report them to immigration authorities (Table 2). Participants acknowledged some individuals might be reluctant to undergo testing for these reasons.

One participant used anonymous testing to alleviate his concerns because he felt more in control of the ways his information was used (Table 2). Another participant explained his family doctor used numbers as a patient identifier on test requisitions, (instead of names), so testing was anonymized for lab processing (Table 2). Participants were open to partner notification by public health if it was done anonymously and did not include identifying information (Table 2).

#### Multi-step process

Participants also described the multi-step process for STI testing as a barrier, which included: making an appointment, traveling to the appointment, waiting in the waiting room –potentially with familiar faces – waiting for test results, and having to return for treatment. Long wait times made the process feel unnecessarily stressful. As one participant described:

“I never wanted to test because it was like, I’d have to make a second appointment, come in, wait in the waiting room, look around like everyone here and then, actually the day I tested, a good friend of mine was in the waiting room and I walked out and he was there and I haven’t spoken to him since, you know, I just haven’t had the courage yet, you know, I saw him once and I just walked by cause I couldn’t, I thought I would break down talking to him. So, the stigma is huge.” (FG1, P6)

Although this participant started by describing the inconvenience of booking an appointment and the stress of waiting, the deeper underlying issue was the stress of compromised anonymity and social stigma.

Recognizing these barriers to delivering STI testing in the clinic, participants supported accessing testing services through online services which would enable requests for lab requisitions and accessing test results online (Table 2). Participants felt this would remove the unnecessary step of the initial clinic visit and streamline follow-up by timely delivering communication of negative test results. They also felt online platforms could provide information about the steps involved in STI testing, including the follow-up of positive test results (Table 2).

### Interactions with healthcare providers

#### Non-stigmatizing, sensitive language & care

Participants reported multiple examples of negative interactions with healthcare professionals that created barriers to regular bacterial STI testing. In one participant’s experience, not all healthcare providers know which tests to offer (Table 3). Many participants spoke about perceived and enacted stigma, where they were concerned about and experienced being judged negatively by a healthcare provider (Table 3). Some healthcare providers also demonstrated transphobia by denying health services (Table 3) or lacked sensitivity by asking questions about gender identity, unrelated to their medical treatment, such as: “How did you know you were trans?” (FG3, P2). These interactions can be experienced negatively by patients and may hinder individuals from seeking healthcare services subsequently.

**Table 3:** Barriers and recommendations to improve bacterial STI testing: interactions with healthcare providers

Subtheme	Barriers	Recommendations	Supportive Quotations
Non-stigmatizing, Sensitive Language & Care	Perceived and enacted stigma and use of stigmatizing language	Recognize stigma and discrimination present in health services and creating processes that address them to deliver destigmatizing and trauma informed care	“When my friends have gone to other doctors, this is both gay friends, going to, maybe non-Village doctors, or straight friends, just at their GPs, when they ask for these things, half the time they’re told they don’t have them, or they don’t know how.” (FG1, P5 extended) “I feel that it’s very, it’s not a sex-positive place. Like they will like, I don’t want to say slut-shame, but they’ll shame you if you have an STI and they have to understand like, it will happen if you’re having sex with condoms or without condoms. Things, skin-to-skin contact, things can transmit as well. If you test positive, they’ll be like you need to use condoms, you can’t do this anymore, and it’s like, we’re having an open, honest conversation like I’m here to get tested. I’m not neglecting my health.” (FG4, P2) “We also have to recognize intersectionalities when it comes to things like, those who have addictions and mental health issues. Especially when, in the queer scene when party and play is a very big theme, and when you’re on substances, and not to stigmatize substance users at all, but to recognize the reality, that, that might also be a barrier for folks to get proper healthcare or to care, and that leads into addictions, self-esteem, and all of those factors that I think, play a huge part in our community.” (FG2, P1) “I also know however that for members of the African-Caribbean and Black community, getting tested for anything is really not something that they run out of the door and go do. […] There are difficulties in getting people tested. Methods to get that changed, I haven’t the foggiest idea what to tell you. […] You have to break down so many barriers and homophobia, transphobia, the church, perhaps maybe that’s one way, to talk to members of the church and get them to get active in getting people saying go out and get tested?” (FG4, P3) “Yeah, they can deny services that happened to me. […] I needed a checkup for HIV and other testing for my immigration, […] and the man was like, I am uncomfortable with knowing that you have a uterus or a vagina. And he asked me to leave.” (FG3, P2) “It’d be great to be able to go to a place that’s warm and welcoming and you know, […] some place where you feel comfortable that they’re gonna keep your stuff confidential.” (FG4, P3) “[It was the] rapport, yeah, and it was kind of amazing, when I went in the second time, I didn’t think like she’d recognise me, and she kind of gave me a smile and like I gave her a smile, like said, hello, and went in and we were chatting, and it also makes it a lot more easier to like, be honest and upfront and open about all my sexual experiences and then she has more information to make more sound decisions about the tests she’s giving me.” (FG2, P4) “So you walk into a clinic and it’s a trans friendly or it’s a trans clinic, and then, you get greeted and you have a form that allows you to put in what name you preferred to be called and pronouns. And then the doctor greets you and asks, what are you here for, and you say, STI check, and instead of asking, oh are you trans and […] they did get that training.” (FG3, P2) “[…] Even if it’s not like sexual violence, sometimes we just dissociate from those parts of ourselves; […] And so, when, there’s a checkup there, I know that, I get uncomfortable, but it’s probably because like, there is no, like I guess there’s like, that consent like, hey, what do you call your body part? Like, what makes you feel comfortable? You know if there was that check-in.” (FG3, P2)

Healthcare providers should be aware of the stigma and discrimination related to gender, sexuality, and mental health when delivering any health service, including bacterial STI testing (Table 3). Trans participants shared a more inclusive environment and experience can be created and promoted by having a question or form asking for preferred name and pronouns when registering with the clinic. Using preferred name and pronouns can prevent inappropriate and potentially harmful use of legal names and mismatched pronouns (Table 3). They also emphasised institutions can do their part in ensuring healthcare providers are trained in anti-discriminatory practice, so healthcare is accessible to all. “I think it’s important for any sort of care […] if people had some sort of like, […] sensitivity training, […] trans people are not that uncommon. […] And it’s really shocking to hear how unprofessional people are.” (FG3, P3).

Participants described the importance of language throughout the STI testing experience. They recommended use of welcoming and patient-centered language, involving active listening without judgement, and building rapport (Table 3). Compassionate patient-centred care utilizes a destigmatizing approach when discussing sexual health and bacterial STIs. It does not “shame you or make you feel like shit if you test positive for something” (FG4, P2) and recognizes each person’s needs are unique. For example, beginning encounters with “do you need anything to make this a more comfortable experience?” (FG3, P1) sets the foundation for a positive experience, especially in situations associated with stigma. “Some people just feel uncomfortable with certain words,” (FG3, P3) so it is important to have discussions with each person regarding terms to use to describe body parts, which could be done in person or on a clinic registration intake form (Table 3). All suggestions are in line with trauma-informed care and take action to destigmatize sexual health and improve bacterial STI testing.

## Discussion

Gay, bisexual, and other GBM who participated in focus groups in Toronto, Canada, expressed a need for accessible efficient bacterial STI testing services delivered in a welcoming environment. Participants recommended STI testing delivery should use a gender and sexual identity affirming, destigmatizing, and trauma-informed approach to encourage first-time and continued regular testing.

To summarize the ideal STI testing experience from the perspective of participants, men would 1) be able to request STI testing virtually or with a scheduled appointment time in an inclusive and culturally safe clinic space; 2) have a provider that uses non-stigmatizing and trauma-informed approach; 3) have sexual history intake standardized using inclusive language that accounts for the spectrum of experiences to inform tests offered; 4) easily access lab requisition and proceed to any lab to collect specimens; 5) understand next steps for negative and positive test results; 6) have access to test results electronically or be informed of test results in a timely manner; and 7) be assured of confidentiality throughout and especially with contact tracing following positive test results.

Our focus groups were held prior to the COVID-19 pandemic, during a time when STI clinics were operating at full capacity. Healthcare providers in Toronto and elsewhere have reported insufficient consultation time as a prevalent barrier to delivering bacterial STI testing [13, 22, 23]. Our focus group participants, similar to healthcare providers, recommended simplifying testing procedures to increase uptake and enable more frequent bacterial STI testing [23]. Participants specifically discussed the need for streamlined services that minimize wait times and use of waiting rooms, along with accessible locations and hours. These recommendations are in keeping with findings in a recent scoping review identifying attributes of HIV and STI testing services preferred by GBM in high income countries [24]. GetCheckedOnline in British Columbia, Canada, the Prelib clinic in Montreal, Canada and the Dean Street Express Service in London, United Kingdom are models of successful online STI testing that address these barriers [25–27]. These models decrease time spent in clinics, and sometimes remove the need to attend a clinic altogether, by focusing on collecting samples, many of which are self-collected. Similarly, since the COVID-19 pandemic, primary care providers in Toronto now offer virtual clinics, using telehealth to provide access to services. This has created a streamlined testing experience involving a phone or video call appointment with a healthcare provider, followed by an electronically forwarded lab requisition to proceed with bacterial STI testing.

Additional ways to streamline testing in primary care and sexual health clinic settings include incorporating self-collection of samples by patients. This strategy was acceptable to men [28–31], has concordance with clinician-collected specimens [29, 32–34], and increases bacterial STI testing service uptake [35]. Similarly, participants supported having allied healthcare providers, such as nurse practitioners and nurses. This strategy was also endorsed by Toronto primary care providers [23] and is often used in sexual health clinics and creates another opportunity to increase capacity to facilitate uptake and more frequent STI testing.

Participants clearly highlighted safe inclusive clinic spaces that provide culturally appropriate services creates a positive testing experience that will promote initial uptake and repeated STI testing. Clinic-based testing environments can destigmatize STI testing by creating welcoming, inclusive, identity affirming spaces, so participants are encouraged to enter the clinic [18]. Including posters and education material that reflect all sexual and gender identities in multiple languages can create more inviting spaces. Clinics offering gendered healthcare services, such as separate women/trans and men/trans hours, should clarify which trans-gender groups are being combined with which cis-gender groups. To reduce stigma regarding STIs and sexual practices, sex positive and pleasure-based approaches can be integrated into the design of clinic environments [36, 37].

Participants also discussed differences in delivery of bacterial STI testing between clinics and suggested standardization. Although Canadian guidelines inform bacterial STI testing, there can be differences in guideline application by proficiency [38]. For example, in surveyed Toronto healthcare providers, those in sexual health clinics or those who saw a higher volume of GBM were more likely to order oral and rectal swabs for testing and less likely to forget to offer testing or be uncomfortable discussing sexual health and testing [23]. It is debatable whether standardized sexual history questions are really appropriate given the range in variability of lived sexual and sexuality experiences. Taking a trauma informed approach, with appropriate, clear, and inclusive language, can facilitate comprehensive collection of sexual health information in a safe way, while ensuring tests ordered are appropriate. It also creates an opportunity for transparency to clarify what is being order and why—information participants felt could be empowering. Computer assisted self-interview for risk assessment is one strategy for standardizing collection of information acceptable to patients and healthcare providers [39]. Furthermore, outlining steps for follow-up, how positive test results will be handled, and ensuring confidentiality in public health reporting is essential in creating continuity of care for STI testing and should be standard practice.

Participants highlighted the role providers play in creating a culturally safe and competent environment, reinforcing the robust evidence base of stigma in interactions in healthcare clinics [18]. Stigma is associated with intersecting social identities and practices (such as same-sex sexual practices, gender and gender identity, race and ethnicity) and health issues (such as STIs) [40]. Stigma is also multi-level, and spans structural levels (e.g., reduced opportunities and mistreatment in education, employment, healthcare), social levels (e.g. community beliefs, norms and attitudes that devalue LGBT persons), and individual levels, where these negatively internalize and result in shame and self-blame [40]. Understanding these wider contexts of stigma as barriers to engaging with bacterial STI testing, healthcare providers must discuss sexual orientation and practices in an open, sex-positive and affirming way. There may be gaps in how providers believe they practice and how patients experience that practice—is it truly patient-centered and compassionate? Providers, institutions and service users can evaluate whether services are delivered using a destigmatizing, anti-discriminatory and trauma informed framework [41, 42].

We intentionally sought gay, bisexual, and other men who have sex with men who were comfortable discussing sexual health and STIs. As we recruited men from the urban downtown centre of Canada’s largest city, we may not have captured the views of men who are less comfortable with discussing their sexuality, or do not self-identify as a man who has sex with men, or who live in more suburban, exurban, or rural areas. Barriers to accessing bacterial STI testing for these men may differ and, consequently, our findings may not be directly transferable. Nevertheless, the solutions suggested, which involve providing patient-centred, lesbian, gay, bisexual and trans (LGBT) affirming care with more accessible opportunities to test, including online, remain promising solutions with high transferability. Given the nuanced experience of the clinical environment in our findings, future work could consider using geo-spatial qualitative methods [43] to understand the person and place interactions to inform intervention development to improve uptake and more frequent STI testing among their local GBM community.

## Conclusions

Our findings offer concrete and practical solutions to improve existing clinical based services and inform efforts to implement new virtual care strategies such as easier access to lab requisitions and availability of online testing and follow-up information. Variety and choice in STI testing options which increase capacity, along with person-centred, LGBT-affirming care, would enable optimal testing.

## Data Availability

The datasets generated and/or analysed during the current study are not publicly available due to participant privacy but are available from the corresponding author on reasonable request.

## Abbreviations

GBM: Gay, bisexual, and other men who have sex with men
HIV: Human immunodeficiency virus
LGBT: Lesbian, gay, bisexual and trans
PrEP: Preexposure prophylaxis
STI: Sexually transmitted infection
